# Real-world analyses of therapy discontinuation of checkpoint inhibitors in metastatic melanoma patients

**DOI:** 10.1038/s41598-020-71788-z

**Published:** 2020-09-03

**Authors:** Marina Amaral de Ávila Machado, Cristiano Soares de Moura, Kelvin Chan, Jeffrey R. Curtis, Marie Hudson, Michal Abrahamowicz, Rahima Jamal, Louise Pilote, Sasha Bernatsky

**Affiliations:** 1 Department of Medicine, McGill University, Montreal, Canada; 2 Centre for Outcomes Research and Evaluation, Research Institute of the McGill University Health Centre, Montreal, Canada; 3 Sunnybrook Odette Cancer Centre, University of Toronto, Toronto, ON Canada; 4 Department of Medicine, University of Alabama At Birmingham, Birmingham, USA; 5 Jewish General Hospital and Lady Davis Research Institute for Medical Research, Montreal, Canada; 6 Department of Epidemiology, Biostatistics and Occupational Health, McGill University, Montreal, Canada; 7 Centre Hospitalier de L’Université de Montréal, Centre de Recherche du CHUM, Université de Montréal, Montreal, Canada

**Keywords:** Melanoma, Drug therapy

## Abstract

The ‘real-world’ patient population of metastatic melanoma is not fully represented in clinical trials investigating checkpoint inhibitors. We described therapy discontinuation in an unselected population-based cohort of adults with metastatic melanoma who started therapy with pembrolizumab, nivolumab, or nivolumab/ipilimumab from January 2015 to August 2017. Therapy discontinuation was defined as a gap between doses beyond 120 days, and/or initiation of another cancer therapy. We estimated drug-specific rate ratios for therapy discontinuation adjusted for age, sex, comorbidities, health care use, and past cancer therapies. We included 876 metastatic melanoma patients initiating pembrolizumab (44.3%), nivolumab/ipilimumab (31.2%), and nivolumab (24.5%). At 12 months of follow-up, the probabilities of therapy discontinuation were 49.9% (95% confidence interval, CI 43.6–56.5) for pembrolizumab, 58.8% (95% CI 50.5–67.3) for nivolumab, and 59.2% (95% CI 51.7–66.8) for nivolumab/ipilimumab. Stratified analyses based on prior cancer therapy, brain metastases at baseline, and sex showed similar trends. In multivariable analyses, compared with pembrolizumab, patients starting nivolumab (rate ratio 1.38, 95% CI 1.08–1.77) or nivolumab/ipilimumab (rate ratio 1.30, 95% CI 1.02–1.65) were more likely to discontinue therapy. Our findings indicate frequent discontinuations of checkpoint inhibitors at one year. The lower discontinuation associated with pembrolizumab should be confirmed in further studies.

## Introduction

Each year, over 100,000 new cases of melanoma are diagnosed in North America. Since 2007, melanoma incidence has been on the rise, while death rates have slowly improved^1^. In the United States (US), melanoma is the third most prevalent cancer among men and the fifth among woman^2^, and melanoma incidence is higher in men (29.3/100,000 persons per year) than women (17.8/100,000 persons per year)^1^. The survivorship considerably reduces from 99% for localized melanoma to 25% for patients diagnosed with distant metastases (5-year relative survival estimates in the US between 2009 and 2015)^1^.

The advent of checkpoint inhibitors has dramatically changed the landscape of treatment for metastatic melanoma. These agents are so-called because they act on key regulators, called checkpoints, of the immune system to help effectively eradicate cancer cells. In the US, ipilimumab (anti-CTLA-4) was approved in 2011 and pembrolizumab and nivolumab (both inhibitors of the programmed death-1, PD-1, pathway) were launched on the market in 2014. Since 2015, the National Comprehensive Cancer Network (NCCN)’s Clinical Practice Guidelines recommended checkpoint inhibitors as first-line treatment for metastatic melanoma. These guidelines shifted slightly in 2016 when monotherapy with pembrolizumab and nivolumab and combination nivolumab/ipilimumab were considered first-line options, due to improved outcomes with these approaches, while ipilimumab monotherapy was moved to a second-line regimen^3–5^. As a side note, in cases of melanoma with a BRAF V600 mutation, BRAF/MEK inhibitors are also considered first-line treatment for metastatic melanoma^6^.

From 2014 to 2016, checkpoint inhibitors (monotherapies and combination therapies) represented 60% of first-line regimens in patients with advanced melanoma (unresectable stage III or metastatic/stage IV) treated in cancer clinics in the US^7^. A claim database study in the US described increases in the frequency of checkpoint inhibitor treatment from 2% of melanoma patients (stages 0-IV) in 2011 to 50% in 2016; from 2014–2016, checkpoint inhibitors became the most commonly used first-line therapies in metastatic melanoma^8^ due to better clinical results in terms of overall survival and progression-free survival^7,9^. The optimal duration of therapy with checkpoint inhibitors remains unknown^6^ and patients may discontinue treatment due to disease progression and toxicities, and initiation of subsequent-line therapies is common in clinical practice^7,9,10^.

Metastatic melanoma patients included in clinical trials do not represent patients in real-world settings, due to exclusion criteria (e.g. brain metastases, autoimmune disease) or other factors (e.g. patient’s performance status)^11^. Therefore, we used real-world data to describe discontinuation of initial checkpoint inhibitor and other treatment patterns in patients with metastatic melanoma. We focused on the currently recommended first-line regimens: pembrolizumab and nivolumab monotherapies and combination therapy of nivolumab and ipilimumab.

## Results

### Patient characteristics

**Figure 1:**
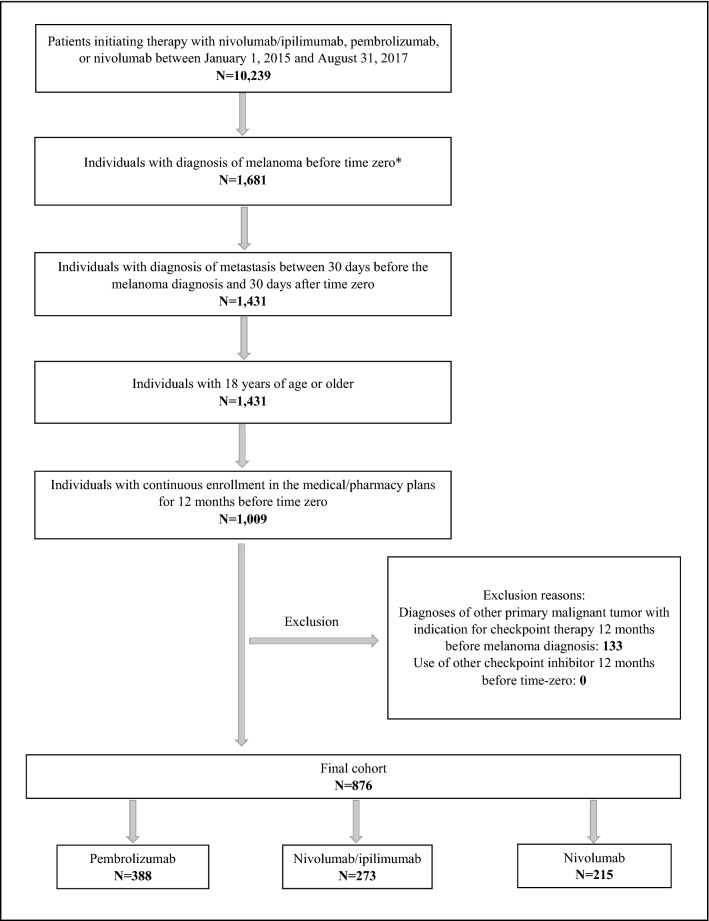
Flow diagram of cohort selection. *Time zero: date of the first claim of pembrolizumab, nivolumab, or nivolumab/ipilimumab.

We included 876 patients with metastatic melanoma who initiated a checkpoint inhibitor, most commonly pembrolizumab (44.3%), followed by nivolumab/ipilimumab (31.2%), and nivolumab (24.5%) (Fig. 1). Overall, 557 (63.6%) of patients were male and the median age at baseline was about 61 years (interquartile range, IQR 52–70). At baseline, employment, comorbidity, previous use of cancer therapy, and number of physician visits showed some heterogeneity across groups. Patients starting therapy with nivolumab/ipilimumab had the highest frequency of full-time employment (55.1%) and commercial health plan type (75.5%) and the lowest frequency of previous use of conventional chemotherapy (1.5%) (Table 1). The variable of race/ethnicity was available only for patients covered by Medicaid (N = 60) and 78.3% of these were Caucasian, 10.0% were black, and the rest were classified as “other”.

**Table 1:** Baseline characteristics of patients included in the cohort (N = 876).

Characteristics	Pembrolizumab N = 388 (44.3%)	Nivolumab N = 215 (24.5%)	Nivolumab/ ipilimumab N = 273 (31.2%)
Male, n (%)	245 (63.1)	136 (63.3)	176 (64.5)
Age in years, median (IQR)	61 (53–72)	63 (55–75)	58 (50–63)
Year of cohort entry, n (%)			
2015	73 (18.8)	40 (18.6)	24 (8.8)
2016	216 (55.7)	125 (58.1)	163 (59.7)
2017	99 (25.5)	50 (23.3)	86 (31.5)
Urban, n (%)^a^	318 (87.4)	170 (88.5)	218 (85.8)
Full-time employment, n (%)^a^	138 (37.9)	58 (30.2)	140 (55.1)
Health plan type, n (%)			
Commercial plans	226 (58.3)	105 (48.8)	206 (75.5)
Medicare	138 (35.6)	87 (40.5)	48 (17.6)
Medicaid	24 (6.2)	23 (10.7)	19 (7.0)
Site of metastases, n (%)			
Brain	100 (25.8)	57 (26.5)	87 (31.9)
Bone	76 (19.6)	54 (25.1)	64 (23.4)
Lymph node	216 (55.7)	104 (48.4)	140 (51.3)
Charlson comorbity index, mean (SD)^b^	1.9 (2.0)	2.5 (2.0)	1.4 (1.5)
Comorbidity, n (%)^b^			
Diabetes	64 (16.5)	44 (20.5)	38 (13.9)
Chronic pulmonary disease	55 (14.2)	37 (17.2)	28 (10.3)
Cerebrovascular disease	45 (11.6)	34 (15.8)	21 (7.7)
Mild liver disease	23 (5.9)	17 (7.9)	32 (11.7)
Congestive heart failure	19 (4.9)	26 (12.1)	11 (4.0)
Renal disease	23 (5.9)	20 (9.3)	8 (2.9)
Previous use of cancer therapy, n (%)^b^			
BRAF/MEK inhibitors	40 (10.3)	24 (11.2)	30 (11.0)
Conventional chemotherapy	24 (6.2)	26 (12.1)	4 (1.5)
Radiotherapy	126 (32.5)	72 (33.5)	95 (34.8)
Melanoma-related surgery	120 (30.9)	56 (26.1)	66 (24.2)
Health care contact, in prior 12 months^b^			
No. oncologist visits, mean (SD)	3.7 (6.1)	5.1 (10.4)	3.8 (7.7)
No. dermatologist visits, mean (SD)	1.8 (3.1)	1.5 (2.3)	1.5 (2.5)
No. other physician visits, mean (SD)	18.8 (15.9)	21.5 (14.8)	15.3 (11.4)
Patients with emergency dep. visits, n (%)	153 (39.4)	92 (42.8)	111 (40.7)
No. emergency dep. visits, mean (SD)^c^	1.9 (1.8)	1.8 (1.3)	1.7 (1.2)
Patients with hospitalizations, n (%)	172 (44.3)	103 (47.9)	123 (45.1)
No. in-hospital days, mean (SD)^e^	10.9 (13.7)	9.8 (12.6)	8.3 (10.0)
Patients with hospice care, n (%)	25 (6.4)	23 (10.7)	14 (5.1)
No. hospice care days, mean (SD)^f^	18.8 (33.5)	15.4 (23.4)	10.6 (24.0)
Follow-up in days, median (IQR)	289.5 (156.0–465.5)	264.0 (138.0–471.0)	234.0 (120.0–447.0)

*IQR* interquartile range, *SD* standard deviation.

^a^Variables only available for patients covered by commercial plans and Medicare.

^b^Measured at one year before time zero.

^c^Among patients who had emergency department visits.

^e^Among patients who had hospitalizations.

^f^Among patients who used hospice care.

### Therapy discontinuation of checkpoint inhibitors

The estimated median time to therapy discontinuation was 12.1 months (95% confidence interval, CI 11.2–14.0) for pembrolizumab, 9.3 months (95% CI 6.8–11.9) for nivolumab, and 7.9 months (95% CI 5.5–10.6) for nivolumab/ipilimumab. At 6 and 12 months, the Kaplan–Meier estimates of probabilities of therapy discontinuation were lower for patients initiating pembrolizumab compared to the other groups. At 24 months, the differences become less prominent and the confidence intervals wider (Table 2). Throughout follow-up, 57% of patients were censored mainly due to loss of medical or pharmacy coverage and the estimates of discontinuation at 24 months were based on fewer subjects impacting their accuracy. The tendency towards lower therapy discontinuation for pembrolizumab was maintained when patients were stratified on the basis of prior cancer therapy, brain metastases at baseline, and sex (Table 2).

**Table 2:** Kaplan–Meier estimates: median time in months to therapy discontinuation (95% confidence intervals) and probabilities (95% confidence intervals) of therapy discontinuation at 6, 12, and 24 months of follow-up for entire cohort and stratified by prior use of BRAF/MEK inhibitors, presence of brain metastases at baseline, and sex.

Therapy discontinuation	Pembrolizumab	Nivolumab	Nivolumab/ipilimumab
**All patients (N = 876)**			
Median time to discontinuation (months)	12.1 (11.2–14.0)	9.3 (6.8–11.9)	7.9 (5.5–10.6)
6 months	23.6% (19.2–28.7)	37.3% (30.5–45.2)	44.9% (38.2–52.1)
12 months	49.9% (43.6–56.5)	58.8% (50.5–67.3)	59.2% (51.7–66.8)
24 months	73.7% (64.9–81.8)	73.3% (63.8–82.0)	72.0% (63.1–80.3)
**Patients with prior use of BRAF/MEK inhibitors (N = 94)**			
Median time to discontinuation (months)	11.4 (6.8–24.1)	11.9 (4.7–25.7)	5.4 (4.7–NA)
6 months	27.3% (15.2–46.0)	34.7% (17.1–62.0)	50.1% (28.7–76.2)
12 months	53.3% (35.6–73.3)	56.4% (31.2–84.3)	60.1% (35.9–85.0)
24 months	65.0% (45.4–83.8)	56.4% (31.2–84.3)	60.1% (35.9–85.0)
**Patients without prior use of BRAF/MEK inhibitors (N = 782)**			
Median time to discontinuation (months)	12.1 (10.8–15.2)	8.7 (6.7–11.5)	7.9 (5.5–10.6)
6 months	23.1% (18.6–28.5)	37.7% (30.5–46.0)	44.5% (37.6–52.0)
12 months	49.4% (42.7–56.4)	58.9% (50.2–67.7)	59.2% (51.4–67.2)
24 months	74.8% (65.3–83.4)	74.7% (64.8–83.7)	73.1% (63.8–81.7)
**Patients with brain metastases at baseline (N = 244)**			
Median time to discontinuation (months)	11.2 (7.9–13.1)	8.7 (4.9–14.5)	13.4 (5.7–19.6)
6 months	26.8% (18.3–38.3)	43.0% (29.2–59.9)	36.3% (24.5–51.6)
12 months	54.4% (41.5–68.4)	61.5% (45–78.2)	49.0% (34.7–65.4)
24 months	82.1% (64.2–94.4)	71.1% (50.1–89.1)	73.2% (52.3–90.4)
**Patients without brain metastases at baseline (N = 632)**			
Median time to discontinuation (months)	12.6 (11.2–16.1)	9.5 (6.8–13.6)	6.5 (5.4–9.1)
6 months	22.5% (17.7–28.3)	35.8% (28.1–44.9)	47.9% (40.2–56.2)
12 months	48.5% (41.4–56.2)	58.0% (48.5–67.8)	62.6% (54.0–71.2)
24 months	71.8% (61.5–81.4)	73.9% (63.2–83.5)	71.8% (62.3–80.6)
**Men (N = 577)**			
Median time to discontinuation (months)	13.3 (11.4–16.1)	8.7 (6.7–15.2)	8.2 (5.7–13.4)
6 months	21.2% (16.0–27.7)	36.6% (28.1–46.7)	41.7% (33.8–50.6)
12 months	48.2% (40.1–57.0)	56.8% (46.4–67.6)	55.2% (46.1–64.7)
24 months	73.6% (62.2–83.9)	70.3% (57.6–82.0)	68.6% (57.5–79.2)
**Women (N = 319)**			
Median time to discontinuation (months)	11.2 (8.9–13.6)	10.1 (5.5–12.4)	6.0 (4.8–9.1)
6 months	27.3% (20.3–36.1)	38.5% (27.7–51.7)	51.0% (39.6–63.6)
12 months	52.4% (42.9–62.7)	61.6% (48.3–75.1)	67.2% (54.2–79.6)
24 months	73.2% (60.0–84.9)	77.7% (63.1–89.5)	79.0% (63.9–90.9)

*NA* not available.

In the multivariable analysis, compared with pembrolizumab monotherapy, patients starting nivolumab monotherapy were more likely to discontinue therapy (adjusted rate ratios, RR 1.38, 95% CI 1.08–1.77) as well as patients starting combination therapy with nivolumab/ipilimumab (adjusted RR 1.30, 95% CI 1.02–1.65) (Table 3). The comparisons between initiators of nivolumab/ipilimumab versus nivolumab monotherapy did not reach statistical significance (Table 3). In the multivariable analysis, sex-by-drug interactions were not significant (*p* > 0.40 for all comparisons between drug groups).

**Table 3:** Rate ratios (95% confidence intervals) for therapy discontinuation (N = 876).

Comparisons	Crude rate ratio	Adjusted rate ratio^a^
Nivolumab/ipilimumab vs. Pembrolizumab	1.37 (1.09–1.73)	1.30 (1.02–1.65)
Nivolumab vs. Pembrolizumab	1.30 (1.02–1.66)	1.38 (1.08–1.77)
Nivolumab/ipilimumab vs. Nivolumab	1.05 (0.80–1.37)	0.94 (0.70–1.25)

^a^Adjusted for sex, age, year of cohort entry, Charlson Comorbidity Index, presence of brain metastases, health plan type, number of outpatient oncology visits, number of outpatient dermatology visits, number of hospitalizations, number of emergency department, and previous use of BRAF/MEK inhibitors or conventional chemotherapy.

### Initiation of another cancer therapy

At 6 months of follow-up, the probability of initiating a second checkpoint inhibitor was higher for patients using nivolumab than pembrolizumab. At 12 months, these groups reached similar estimates (around 17%), while the probability was 8.6% for users of combination therapy. During the same period, the probability of initiating BRAF/MEK inhibitors was 13.3% for patients using combination therapy and 5.7% for users of pembrolizumab (Table 4). Users of both pembrolizumab and nivolumab had lower probabilities (8% and 10%) of cessation of initial therapy at 6 months of follow-up, compared to combination therapy (28%) (Table 4).

**Table 4:** Kaplan–Meier estimates: probabilities (95% confidence intervals) of initiation of another cancer therapy, of cessation of initial therapy, and initiation of treatment with systemic corticosteroid at 6, 12, and 24 months of follow-up (N = 876).

Outcomes	Pembrolizumab	Nivolumab	Nivolumab/ipilimumab
**Initiation of a different checkpoint inhibitor**			
6 months	9.8% (7.0–13.6)	14.8% (10.3–21.0)	4.6% (2.5–8.4)
12 months	17.6% (13.4–22.8)	17.1% (12.1–23.7)	8.6% (5.1–14.2)
24 months	20.5% (15.5–26.9)	18.9% (13.2–26.6)	8.6% (5.1–14.2)
**Initiation of BRAF/MEK inhibitors**			
6 months	4.4% (2.7–7.2)	7.1% (4.1–11.9)	10.4% (7.1–15.1)
12 months	5.7% (3.6–9.0)	9.3% (5.8–14.9)	13.3% (9.2–18.9)
24 months	6.9% (4.1–11.2)	9.3% (5.8–14.9)	14.5% (10.0–20.6)
**Initiation of conventional chemotherapy**			
6 months	2.3% (1.0–5.4)	5.4% (2.8–10.1)	2.1% (1.0–4.4)
12 months	3.9% (1.8–8.2)	5.4% (2.8–10.1)	4.3% (2.3–7.8)
24 months	10.8% (4.5–24.7)	6.9% (3.6–13.0)	7.5% (3.8–14.6)
**Cessation of initial therapy**^**a**^			
6 months	7.8% (5.2–11.5)	9.9% (6.2–15.6)	28.2% (22.4–35.1)
12 months	27.8% (22.2–34.4)	28.1% (20.9–37.2)	37.0% (30.2–44.8)
24 months	53.7% (42.8–65.5)	41.7% (31.7–53.5)	49.7% (40.4–59.9)
**Initiation of systemic corticosteroid**			
6 months	28.0% (22.3–34.8)	22.9% (15.8–32.6)	79.7% (72.2–86.2)
12 months	43.0% (35.8–51.0)	45.7% (35.1–57.9)	83.9% (76.3–90.2)
24 months	62.6% (51.8–73.4)	60.5% (47.0–74.4)	88.3% (79.9–94.3)

^a^Gap between doses beyond 120 days without initiating a new cancer therapy.

### Secondary outcomes

In the combination therapy group, the mean number of ipilimumab doses per patient was 2.9 (standard deviation, SD 1.3) and 40.3% (95% CI 31.2–44.4) of patients received four doses or more of ipilimumab during follow-up. Regarding initiation of treatment with systemic corticosteroids, we observed higher probabilities for users of nivolumab/ipilimumab compared to pembrolizumab or nivolumab at all time-points. At 6 months, the probability was 79.7% for combination therapy and less than 30% for monotherapies (Table 4).

## Discussion

This population-based cohort study used real-world data from US healthcare claims of patients with metastatic melanoma starting therapy with checkpoint inhibitors from January 2015 to August 2017. We observed a peak of new users of checkpoint inhibitors in 2016 and, throughout the study period, most patients started therapy with pembrolizumab monotherapy. We included patients slightly younger than those reported in US statistics for melanoma patients^1^, but similar sex and age distribution of stage III and metastatic melanoma patients included in studies originated from cancer clinics in the US^7,9^.

Our main analysis showed that the median time to therapy discontinuation was 12 months for pembrolizumab, 9 months for nivolumab, and 8 months for nivolumab/ipilimumab. The multivariable analyses showed higher discontinuation rate of nivolumab monotherapy or nivolumab/ipilimumab compared with pembrolizumab, though Kaplan–Meier estimates suggest that from 12 months onwards, all regimens had comparable discontinuation. Previous US data showed a trend towards higher time to discontinuation of anti-PD-1 agents (pembrolizumab or nivolumab monotherapies) compared to combination therapy^7,9^.

Contrasting the finite duration of therapy with ipilimumab (4 doses within 16 weeks), the optimal duration of therapy with pembrolizumab or nivolumab remains a matter of debate. It is unknown what duration of anti-PD-1 therapy is required to generate a sufficient and durable immune response^12,13^. One observational cohort of 185 patients with advanced melanoma who discontinued anti-PD-1 agents (in absence of progressive disease or treatment limiting toxicity) at a median therapy duration of 12 months went on to have 1-year and 2-year progression-free survival rates of 90% and 71%^13^. This supports the notion of durable anti-tumour responses well beyond discontinuation (due immunological ‘memory’ effects) and that discontinuation may not be necessarily a bad outcome^14^.

Some melanoma patients may discontinue checkpoint therapy due to disease progression, in which case switching to a different drug class is recommended^6^. Other patients discontinue due to immune-related adverse events (irAEs) such as arthritis, pneumonitis, colitis, and other syndrome^9,10,15^, which often require corticosteroids. In case of severe or life-threatening irAEs, therapy must be permanently discontinued; for moderate adverse events, therapy may be temporarily suspended and restarted after resolution of the irAE^16^. In our study, compared to monotherapies, patients using nivolumab/ipilimumab were more likely to stop therapy and not initiate another cancer medication, and more likely to receive systemic corticosteroid (probability of 80% at 6 months), suggesting more short-term toxicities. This resembles findings from clinical trials and other observational studies^9,17^. However, combination therapy has been associated with improved response and progression-free survival compared with monotherapy^5^. For that reason, nivolumab/ipilimumab still holds an important role in first-line therapy if patients are willing to accept a higher risk of irAEs (in the absence of comorbidities and autoimmune conditions)^6^.

Although nivolumab and pembrolizumab users in our study had similar probability of cessation of initial therapy, the nivolumab group tended to initiate another cancer therapy earlier, which may have been due to earlier disease progression. Some observational studies show that both anti-PD-1 agents have similar effectiveness^9,18^, although in a recent study, patients on nivolumab had a numerically shorter time to discontinuation/death (11 months) versus pembrolizumab (16 months)^18^. In our investigation, pembrolizumab was more commonly used (n = 388, 44.3%, 95% CI 41.0–47.6%) than nivolumab (n = 215, 24.5%%, 95% CI 21.7–27.4%). This may suggest a slight preference for pembrolizumab versus nivolumab on the part of patients, clinicians, or payers, though we are unable to test this hypothesis. Other factors affecting therapy choice may include dosing schedule (every 3 weeks for pembrolizumab versus every 2 weeks for nivolumab).

We performed subgroups analyses stratified by prior use of BRAF/MEK inhibitors and presence of brain metastases at baseline. Few patients (11%) used BRAF/MEK inhibitors before initiation of checkpoint inhibitors and less than 30% of patients had brain metastases at baseline, a feature with extremely poor prognosis and short overall survival, even with checkpoint inhibitor treatment^19–21^. Our stratified analyses were not conclusive regarding a distinction of therapy discontinuation between patients with and without prior use of BRAF/MEK inhibitors and between patients with and without brain metastases. We were also interested in testing sex differences considering it is well known that adult females have stronger innate and adaptive immune responses than males^22^, which theoretically could translate into differential effects of immune system checkpoint inhibitors. A previous retrospective study found that only 12% of male metastatic melanoma discontinued therapy (pembrolizumab or nivolumab) due to immune-related adverse events, compared to 23% of women^23^. In our study, we were unable to detect sex differences in the time to therapy discontinuation.

Our study has considerable strengths because we analysed a large cohort of unselected patients and presented real-world therapy discontinuation of checkpoint inhibitors, which was not a focus of prior studies^8,24^. Our study also has some potential limitations. First, patients can enter and leave the register in MarketScan databases as their medical insurance changes, creating challenges in both the assessment of early drug exposures and the follow-up patients, but this is unlikely to be differentially related to each initial therapy. Second, even though we analysed three first-line therapies, and controlled for demographics, comorbidities, and past drug use, it is possible that residual confounding by indication or disease severity was still present. Finally, given the scope of our study, we could not determine reasons for therapy discontinuation or whether such event happened at the expense of partial or complete clinical response.

In conclusion, these real-world data demonstrated that a large proportion of patients discontinued initial therapy with pembrolizumab, nivolumab, or nivolumab/ipilimumab before two years and that difference in therapy discontinuation was very small or not detectable between patients initiating those therapies, but with trends for lower discontinuation with pembrolizumab. Our results also indicated that at least one-half of patients initiated therapy with systemic corticosteroid and one-quarter switched to another cancer therapy at one year of follow-up. We also observed a low completion of ipilimimab induction regimen in users of combination therapy. We emphasize the need for further investigations to confirm these findings and to evaluate other aspects of treatment, including subsequent-line therapies.

## Methods

### Data sources

We used data from the IBM MarketScan Commercial Claims Database, Medicare Supplemental Database, and Multi-State Medicaid Database for the period of January 1, 2011 and December 31, 2017. These databases include information on physician office visits, inpatient services, hospital stays, prescription drugs, and enrollment data.

### Study population

We included adults (aged ≥ 18) with at least one claim for ipilimumab, pembrolizumab, or nivolumab initiated between January 1, 2015 and August 31, 2017. Date of the first relevant drug claim was defined as time zero. Individuals were required to have at least one outpatient or inpatient melanoma diagnostic claims code before time zero, plus one metastasis outpatient or inpatient diagnostic claims code between 30 days before the melanoma diagnosis and 30 days after time zero (Table 5). We further required continuous enrollment in the medical and pharmacy plans for 12 months before time zero. We excluded individuals who had diagnoses of other primary malignant tumor with an indication for checkpoint therapy 12 months before melanoma diagnosis (Table 5), those who used any checkpoint inhibitors any time before time zero (minimum 12 months before) so as to identify new users, and those who started two or more checkpoint inhibitors and MEK inhibitors at time zero, except nivolumab/ipilimumab (one of the regimens considered in the present study).

**Table 5:** List of International Classification of Diseases (ICD) codes used in the study.

Disease	ICD-9	ICD-10
**Inclusion criteria**		
Malignant melanoma of skin	172, V10.82	C43, Z85.820
Metastasis	196, 197, 198	C77, C78, C79
**Exclusion criteria—malignant tumors**		
Gastric or gastroesophageal	150, 151	C15, C16
Urothelial carcinoma	188, 189.2, 189.3	C65, C66, C67
Hodgkin lymphoma	201	C81
Head and neck squamous cell cancer	173.02, 173.12, 173.22, 173.32, 173.42	C44.02, C44.12, C44.22, C44.32, C44.42
Hepatocellular carcinoma	155	C22
Renal cell carcinoma	189.0	C64
Lung cancer	162	C34
**Covariates**		
Brain metastases	198.3	C79.3
Bone metastases	198.5	C79.5
Lymph node metastases	196	C77

The claims for ipilimumab, pembrolizumab, or nivolumab were identified using Healthcare Common Procedure Coding System (HCPCS) codes from outpatient claims (C9284 and J9228 for ipilimumab; C9027 and J9271 for pembrolizumab; C9453 and J9299 for nivolumab). We defined three groups based on initial drug therapy: pembrolizumab, nivolumab, and nivolumab/ipilimumab. The latter was defined as treatment with two claims for each drug within 14 days of each other.

### Study outcomes

Our primary outcome was therapy discontinuation of initial checkpoint inhibitor defined as a gap between doses beyond 120 days and/or initiation of another cancer therapy (different checkpoint inhibitor, BRAF/MEK inhibitors, or conventional chemotherapy). The date of therapy discontinuation corresponded to 120 days after the end of the last claim for the initial therapy or the date of initiation of a new cancer therapy, whichever came first. Regarding the combination therapy (nivolumab/ipilimumab), we considered therapy discontinuation for the nivolumab only because ipilimumab is given in combination with nivolumab for regimen of four doses within 16 weeks from the initial dose and thereafter nivolumab continues as monotherapy. We also analysed the outcomes of initiation of different checkpoint inhibitor, BRAF/MEK inhibitors, or conventional chemotherapy separately, and an outcome of cessation of initial therapy defined as a gap between doses beyond 120 days without initiating a new cancer therapy. Secondary outcomes were the number of total ipilimumab doses during follow-up to verify the completion of the combination regimen and initiation of systemic therapy with corticosteroids (oral or intravenous).

### Covariates

Study covariates included socio-demographic variables measured at time zero, namely sex, age, calendar year of checkpoint therapy initiation, metastases (brain, bone, and lymph node), health plan type (commercial, Medicare, or Medicaid), residence (urban vs. rural area, available for commercial and Medicare only), employment status (commercial and Medicare only), and race/ethnicity (Medicaid only). Other covariates were measured during the year before time zero and included the Charlson Comorbidity Index (excluding codes for melanoma and metastatic solid tumor); use of another cancer therapy such as BRAF/MEK inhibitors, conventional chemotherapy, radiotherapy, and melanoma-related surgery; contact with health system measured by emergency department visits, hospitalizations, outpatient physician visits, and hospice care.

### Statistical analysis

We performed descriptive analyses of patient baseline characteristics stratified by the different checkpoint inhibitor groups. In the analysis, patients were followed from the date of the first claim of checkpoint inhibitor (time zero) until either the occurrence of an event of interest or censored in case of death (only in-hospital deaths were available), loss of medical and pharmacy coverage, or end of study period (December 31, 2017). We used the Kaplan–Meier method to estimate median time to discontinuation of each checkpoint therapy and probabilities of discontinuation at 6, 12, and 24 months for the entire cohort and stratified by previous use of BRAF/MEK inhibitors (another first-line option for metastatic melanoma), presence of brain metastases at baseline, and sex. Similarly, we estimated probabilities of initiating a (1) different checkpoint inhibitor, (2) BRAF/MEK inhibitors, (3) conventional chemotherapy, and (4) systemic corticosteroids at 6, 12, and 24 months for the entire cohort. We applied Poisson regression with robust variance to estimate the adjusted rate ratios with 95% CIs to compare the primary outcome of therapy discontinuation for each checkpoint inhibitor. RR’s were adjusted for sex, baseline age, year of checkpoint therapy initiation, presence of brain metastases at baseline, health plan type, and a set of covariates measured during the year before time zero: Charlson Comorbidity Index, previous use of BRAF/MEK inhibitors or conventional chemotherapy, number of outpatient oncology visits, number of outpatient dermatology visits, number of hospitalizations, and number of emergency department. Healthcare use might be related to impairment severity and disability, and in the absence of clinical measures, we used those variables to adjust the model. In addition, we tested two-way sex-by-drug interactions in the Poisson model to explore if the comparisons of therapy discontinuation of different checkpoint inhibitors differed between women and men. In the case of a significant interaction (*p* < 0.05 for the multivariable model-based Wald test), the corresponding analyses were repeated separately for men and women. All analyses were performed using SAS version 9.4 (SAS Institute Inc, Cary, NC, USA).

## Data Availability

IBM MarketScan Commercial Claims Database, Medicare Supplemental Database, and Multi-State Medicaid Database are not in the public domain but are available to researchers at a cost.
